# Photo Quiz: An adolescent male with fever, rash, and polyarthralgia

**DOI:** 10.1128/jcm.01228-25

**Published:** 2026-04-08

**Authors:** Benjamin von Bredow, Bobby L. Boyanton

**Affiliations:** 1 Department of Pathology, Oakland University William Beamont School of Medicine 159878, Rochester, Michigan, USA; 2 Department of Pathology & Laboratory Medicine, Corewell Health William Beaumont University Hospital 21818, Royal Oak, Michigan, USA; 3 Department of Pathology & Laboratory Medicine, University of Arkansas for Medical Sciences 12215, Little Rock, Arkansas, USA; 4 Department of Pathology & Laboratory Medicine, Arkansas Children’s Hospital, Little Rock, Arkansas, USA; Mayo Clinic Minnesota, Rochester, Minnesota, USA

## PHOTO QUIZ 

**Fig 1:**
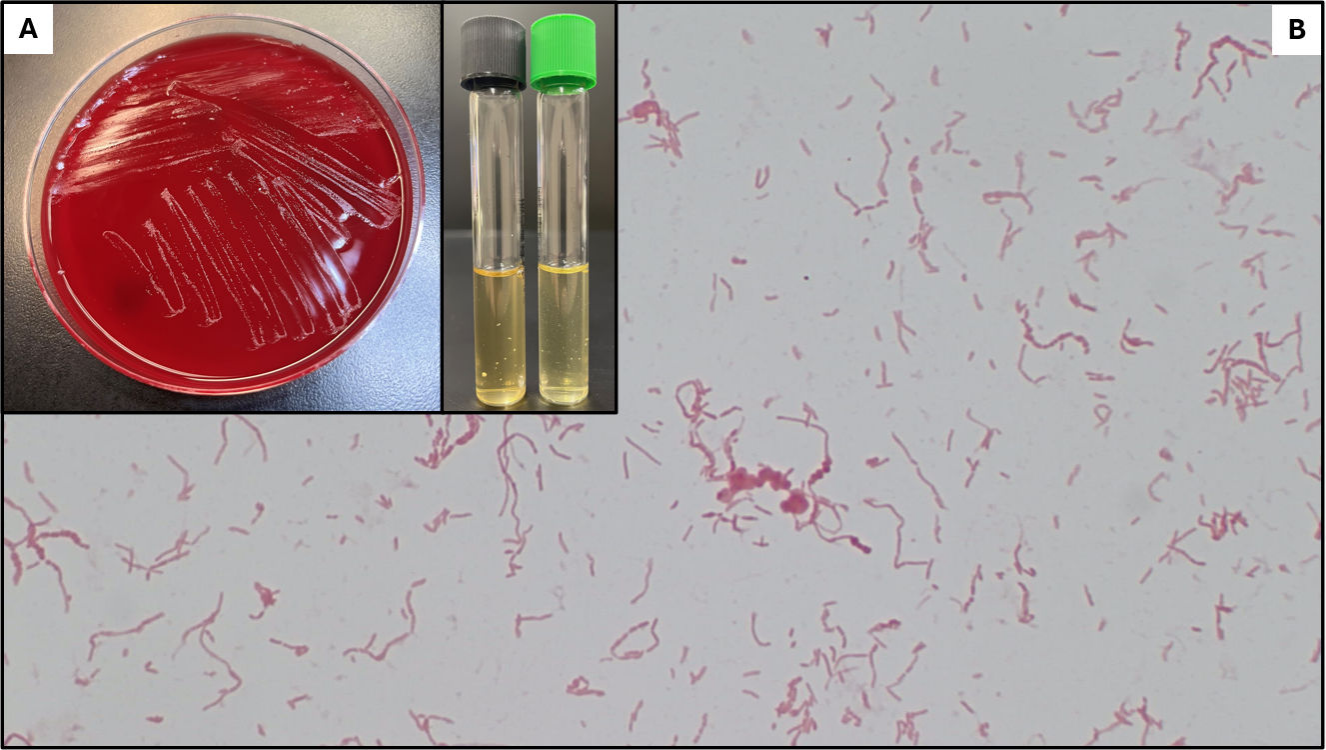
Anaerobic subculture from positive blood cultures. (**A**) Left panel—CDC anaerobic agar at 72 h incubation; right panel—thioglycollate broth (black cap) and tryptic soy broth (green cap) at 7 days incubation, demonstrating “bread crumb” growth. (**B**) Gram stain (500× oil) of culture growth showing gram-negative bacilli in short-to-long individual and intertwined chains with bulbar swellings.

A teenage male presented to the emergency department with fever (38.9°C), chills, nausea, fatigue, arthralgias, and rash of 1-day duration. He visited the beach a day prior but continuously used sunscreen, denied being stung/bitten by sea life, and did not experience vomiting, sore throat, or diarrhea. The physical examination was significant for a maculopapular rash on the torso and extremities, including the palms and soles. He lived at home with his mother and eight siblings. He frequently interacted with family pets (i.e., five finches, three dogs, two turtles, two rats, and one parrot) but denied being bitten. A complete blood count showed a white blood cell count of 22.0 bil/L (normal 4.5–13) with a predominance of neutrophils (92%), hemoglobin of 115 g/L (normal 135–175), and platelet count of 300 bil/L (150–400). Erythrocyte sedimentation rate and C-reactive protein were 35 mm/h (normal 0–15) and 106 mg/L (normal <8.0), respectively. Monospot and Group A Strep antigen were negative. Intravenous ceftriaxone was administered following collection of two sets of blood cultures. A pleomorphic gram-negative bacillus was recovered from both sets of aerobic bottles (BD BACTEC; Becton Dickinson, Franklin Lakes, NJ, USA) following 3 days of incubation. Scant subculture growth was obtained on sheep blood and chocolate agar after 72 h of aerobic incubation. Abundant subculture growth was obtained on CDC anaerobic sheep blood agar following 72 h of anaerobic incubation. Colonies were non-hemolytic, tiny, circular, gray, and smooth (Fig. 1A) and negative for catalase, oxidase, and indole. Cultured isolates demonstrated gram-negative bacilli arranged as single cells, short chains, or long chains that intertwine with bulbar swellings (Fig. 1B). Identification was made by matrix-assisted laser desorption/ionization time-of-flight mass spectrometry (MALDI-ToF; Bruker, Billerica, MA, USA).

## ANSWER TO PHOTO QUIZ

The patient was infected with *Streptobacillus moniliformis*. He was discharged on hospital day 3 and completed 14 days of amoxicillin with infection resolution. He and his family were counseled about infectious risks associated with pets (rats most likely being the source of his infection) and educated on proper hand hygiene (the most likely source of organism acquisition).

There are five species in the genus *Streptobacillus*, namely, *S. canis*, *S. felis*, *S. moniliformis*, *S. notomytis*, and *S. ratti* (1). *S. actinoides* was initially described in 1982; however, as of 2024, its nomenclatural status has yet to be established (1). Although initially described in 2014, *S. hongkongensis* was reclassified as *Pseudostreptobacillus* (novel genus) in 2020 (2, 3). Regarding human infection, *S. moniliformis* is the predominant infectious agent globally and the focus of this manuscript (1, 4). Outside of North America (primarily Asia), *Spirillum minus* is a less commonly implicated cause of rate bite fever and discussed elsewhere (4–6).

The term “rate bite fever (RBF)” not only describes pathogen acquisition following a rodent bite or scratch, but also includes close contact, so-called “rodent kissing” (7). “Haverhill fever” was coined in 1926 and described organism acquisition via contaminated milk consumption, but now signifies pathogen acquisition via ingestion of any rodent excrement-contaminated food/liquid (8). To date, 29 countries have documented cases of RBF, with the highest number reported in the United States (*n* = 92), United Kingdom (*n* = 20), Japan (*n* = 12), France (*n* = 10), Canada (*n* = 9), Australia (*n* = 6), Spain (*n* = 6), and Germany (*n* = 4) (1). In the United States, more than 2 million animal bites occur annually; rats are responsible for 1% (1, 6). Referrals to emergency departments for RBF are estimated at 0.33 per 1,000,000 individuals, with 60% of diagnosed cases requiring hospitalization for management, and more than 30% of these occur in children under 15 years of age (1). The true incidence of RBF is likely underrepresented since isolating and identifying the organism are challenging, and it is not a reportable disease. Maintaining rats as family pets is commonplace, and colonization rates by *S. moniliformis* in rats can be approximately 100% (5, 6, 9). The risk of infection following a rat bite is 10%, with a mortality of 13% if left untreated (5, 6).

Clinical symptoms associated with *S. moniliformis* infection typically begin within 1–2 weeks after exposure with abrupt onset of fever, rigors, myalgias, and vomiting. Over the next few days, patients develop migratory polyarthralgia (usually knees and ankles) that can last for years and a rash (maculopapular, petechial, purpuric) that spreads to the extremities, including the palms and soles, that are tender and can desquamate (5). Fever can relapse at irregular patterns for months. Untreated patients may develop endocarditis, myocarditis, pericarditis, pneumonia, and meningitis (5, 10). *S. moniliformis* is susceptible to penicillin, ampicillin, cephalosporins, erythromycin, clindamycin, tetracycline, imipenem, and vancomycin (1, 5, 6). Most isolates are resistant to aminoglycosides, norfloxacin, nalidixic acid, polymyxin B, and trimethoprim/sulfamethoxazole (1). Patients with endocarditis require therapy with high-dose penicillin G in combination with streptomycin or gentamicin to enhance activity against cell wall-deficient “L-forms” of *S. moniliformis* (5)

*S. moniliformis* is a non-motile, pleomorphic, filamentous, gram-negative bacillus that displays bulbar swellings (1, 5). Being fastidious, it requires a microaerophilic environment and blood or serum for cultivation. On agar, colonies may take 2 to 7 days to appear and are small, circular, convex, grayish, and glistening (1, 5). Growth is inhibited by sodium polyanethol sulfonate, an additive commonly used in blood culture bottles. Colonies appear as “breadcrumbs” or “puffballs” when cultivated in tryptone soy broth (Fig. 1B). It is biochemically inert and negative for catalase, indole, and oxidase (1, 5). MALDI-ToF MS has largely replaced conventional identification methods; 16S rRNA gene sequencing and/or Fourier-transformed-infrared spectroscopy are alternate identification options (10). For our patient’s isolate, the U.S. Food and Drug Administration (FDA)-approved Biotyper CA software (version 3.2, build 16) provided a match to *Citrobacter amalonaticus* complex with an unacceptable probability score of 1.25. Repeat analysis was performed in triplicate using the accompanying MBT Compass HT RUO Software (version 5.1.420.61), which yielded an identification of *S. moniliformis* with a mean score of 2.49 +/− 0.3. Although identification probability scores for other *Streptobacillus* species were not listed in our case, it has been shown that other *Streptobacillus* species (i.e., *S. ratti, S. canis, S. notomytis,* and *S. felis*) can be reliably differentiated using MALDI-ToF MS (1).

The timely diagnosis of RBF relies upon a thorough physical examination and history to elucidate risk factors for disease acquisition. Providers should seek advice from infectious diseases physicians and clinical microbiologists, and most importantly, inform their clinical microbiology laboratory to ensure that appropriate cultivation techniques are employed to optimize pathogen recovery. When *S. moniliformis* is suspected, laboratories should consider employing SPS-free media, such as trypticase soy broth, and extending incubation times, particularly for cultures and subcultures on solid media (1, 5). The unique Gram stain morphology can also help guide subculture techniques if observed in a positive blood culture or primary Gram stain. As demonstrated in our case, CDC anaerobic sheep blood agar enhanced pathogen recovery and could be utilized in suspected RBF cases. For laboratories using manual processes, blood culture bottles should be incubated at 35–37°C and visually inspected daily for signs of microbial growth (e.g., turbidity, hemolysis, and gas bubbles). Regardless of visual clues, best practice dictates aseptically removing an aliquot from each bottle daily for Gram stain and subculture. Recovery of *S. moniliformis* is enhanced by employing tryptone soy agar/broth and Columbia agar with 5% defibrinated sheep blood incubated under aerobic, aerobic with 5% CO_2_, and if possible anaerobic conditions (2, 10).
